# Comparison of copper and zinc in vitro bioaccessibility from cyanobacteria rich in proteins and a synthetic supplement containing gluconate complexes: LC–MS mapping of bioaccessible copper complexes

**DOI:** 10.1007/s00216-015-9162-8

**Published:** 2015-11-23

**Authors:** Justyna Wojcieszek, Katarzyna Witkoś, Lena Ruzik, Katarzyna Pawlak

**Affiliations:** Chair of Analytical Chemistry, Faculty of Chemistry, Warsaw University of Technology, Noakowskiego 3, 01-664 Warsaw, Poland

**Keywords:** Spirulina, Inductively coupled plasma mass spectrometry, Capillary liquid chromatography–electrospray mass spectrometry, Bioaccessibility, Copper complexes, Pepsin peptide map

## Abstract

An analytical procedure was proposed to estimate bioaccessibility of copper and zinc in Spirulina Pacifica tablets with respect to that of copper and zinc in gluconate complexes. Spirulina is the common name for diet supplements produced primarily from two species of cyanobacteria, namely *Arthrospira platensis* and *Arthrospira maxima.* Spirulina tablets are an excellent source of proteins, vitamins and minerals. To obtain information about the bioavailability of these elements, an in vitro bioaccessibility test was performed by application of a two-step protocol which simulated the gastric (pepsin) and intestinal (pancreatin) digestion. The species obtained were investigated by size exclusion chromatography on a chromatograph coupled to a mass spectrometer with inductively coupled plasma (SEC–ICP–MS) and an on-capillary liquid chromatograph coupled to an electrospray mass spectrometer (μ-HPLC–ESI–MS). Both copper and zinc were found to be highly bioaccessible in Spirulina tablets (90–111 %) and those containing gluconate complexes (103 % for Cu and 62 % for Zn). In Spirulina tablets, copper was found to form two types of complex: (1) polar ones with glycine and aspartic acid and (2) more hydrophobic ones containing amino acids with cyclic hydrocarbons (phenylalanine, histidine, proline and tyrosine). Zinc and copper were also proved to form complexes during the digestion process with products of pepsin digestion, but the stability of these complexes is lower than that of the complexes formed in Spirulina. The results proving the involvement of proteins in the enhancement of copper and zinc bioaccessibility will be useful for the design of new copper and zinc supplements.

**Electronic supplementary material:**

The online version of this article (doi:10.1007/s00216-015-9162-8) contains supplementary material, which is available to authorized users.

**Graphical Abstract:**
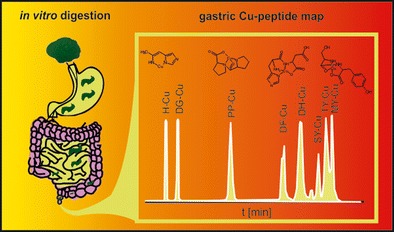
LC–MS mapping of bioaccessible copper complexes from cyanobacteria

## Introduction

Evaluation of bioavailability of macro minerals and trace elements in food and food supplements is important from the nutritional, toxicological and pharmacological points of view. Zinc acts as an intracellular signalling ion which permits communication between cells by converting extracellular stimuli into intracellular signals and controlling intracellular actions. Thus, alteration of zinc homeostasis and dysfunction in the signalling function of zinc may cause pathogenesis [1, 2]. Zinc also plays a substantial role in enhancement of reproductive abilities and it is also required for optimal cellular function of more than 300 different enzymes [3]. Copper is another important trace element which is needed for correct activity of many Cu-dependent enzymes such as lysyl oxidase, cytochrome c oxidase, tyrosinase, dopamine β-hydroxylase, peptidyl glycine α-amidating monooxygenase, monoamine oxidase, ceruloplasmin, superoxide dismutase (SOD) and all the enzymes that act as an antioxidant defence system. The homeostasis of both enzymes and metals responsible for their activation is necessary for human beings [4].

Copper and zinc are trace elements essential for the biochemical functions of photosynthetic organisms [5]; these organisms can be used as supplementary sources of copper and zinc without the risk for the disorder of the metal equilibrium. Spirulina is a good example of a “green” diet supplement produced primarily from two species of cyanobacteria, namely *Arthrospira platensis* and *Arthrospira maxima* [6, 7]. *Arthrospira* has been used since ancient times because of its high nutritional value [8, 9]. It is an excellent source of proteins (60–70 % of dry weight) [10], vitamins and minerals [11]. Cyanobacteria are also a rich source of provitamin A (β-carotene), minerals, carotenoids and phycocyanins [12, 13]. *Arthrospira* can produce polyunsaturated fatty acids such as linoleic acid (C18:2) and γ-linolenic acid (GLA, C18:3) [14, 15].

Although great progress has been made in analytical instrumentation, the speciation analysis of zinc and copper in food has not been particularly successful [14]. Investigation of the affinity of copper toward peptides in spinach leaves [16] and samples of milk or infant formulas was carried out by means of high-performance chromatography or capillary electrophoresis coupled to different spectrometric detectors [17]. Low stability of the complexes and especially the reactivity of zinc and copper inducing redox reactions in the electrospray spray chamber have been a bottleneck for quantitative and qualitative analyses. The most frequent reactions are dissociation of most reactive groups leading to oxidation (e.g. cysteine and glutathione), decarboxylation and dehydration during collision-induced dissociation (CID) fragmentation in alkaline media [18]. The involvement of metal ions in the formation of intramolecular hydrogen bonding and formation of high molecular clusters [19, 20] can be, to some extent, suppressed by addition of organic solvent [21, 22].

The aim of this study was the estimation of zinc and copper bioaccessibility in Spirulina tablets by in vitro simulation of gastrointestinal digestion compared to that from a synthetic diet supplement containing copper and zinc gluconate [23, 24]. Another objective was the identification of the post-digestion copper and zinc complexes responsible for higher bioavailability of metals, which is of interest for the design of synthetic supplements [25].

The main novelty of this study was the application of μ-RPLC–ESI–MS/MS to generate maps of copper/zinc complexes with short peptides obtained via enzymatic digestion with pepsin (gastric digestion) and pancreatin cocktail (gastrointestinal digestion) instead of trypsin (typical for a bottom-up approach in proteomics where longer peptides are required for univocal identification of proteins). This is, to the best of our knowledge, the first attempt to obtain metal-specific gastric and gastrointestinal peptide maps.

## Experimental

### Instrumentation

#### SEC–ICP–MS

Chromatographic separations were performed using an Agilent 1100 gradient HPLC pump (Agilent Technologies, Waldbronn, Germany) as the sample delivery system. All connections were made of PEEK tubing (0.17 mm i.d.). An Agilent 7500a ICP mass spectrometer (Tokyo, Japan) was used as an element-specific detector for quantification of metal content in Spirulina tablets and as an on-line HPLC detector. A Ni/Cu-skimmer was installed in the interface; the position of the torch and the nebulizer gas flow were adjusted daily with special emphasis on decreasing the level of CsO^+^ below 0.2 % in order to minimize the risk of polyatomic interferences caused by oxides.

The screening for the metal complexes was performed by means of size exclusion chromatography on a chromatograph coupled to ICP–MS. Copper and zinc species were eluted from a SEC Superdex200 10/300GL (GE Healthcare Life Sciences) column with 30 mM Tris–HCl buffer (pH 7.4) as the mobile phase.

#### μ-HPLC–ESI–MS/MS

Capillary HPLC–ESI–MS/MS analyses were performed with an Agilent 1200 series chromatograph (Agilent Technology, Waldbronn, Germany) equipped with a binary capillary pump, degasser, autosampler, thermostatically controlled column compartment and capillary Zorbax SB C18 column coupled to an electrospray ionization triple quadrupole mass spectrometer (Agilent 6460 Triple Quad LC/MS, Agilent Technologies, Santa Clara, CA, USA). All the operations, data acquisition and analysis were processed by MassHunter Software (Agilent Technology, USA). Operational parameters are summarized in Table S1 (see Electronic Supplementary Material).

A Bandelin Sonorex model 1210 ultrasonic bath (Germany) and MPW model 350R centrifuge (MPW Warsaw, Poland) were used for extraction procedures. Sample mineralization was performed with a Speedwave®4 microwave digestion system (Berghof, Germany).

### Reagents

The majority of reagents used were of analytical reagent grade purchased from Sigma–Aldrich (Sigma–Aldrich, Buchs, Switzerland). Formic acid of LC/MS purity was purchased from Fisher Scientific (Fair Lawn, NJ, USA). Methanol (LC–MS grade) was purchased from POCH (Gliwice, Poland). Pepsin from porcine gastric mucosa and pancreatin cocktail were of biological grade (from Sigma-Aldrich, Buchs, Switzerland). Water (18 MΩ cm) prepared with a Milli-Q system (Millipore Elix 3, Millipore, Saint-Quentin, France) was used throughout.

### Sample preparation

#### Sample mineralization toward metal determination in supplements

The Spirulina tablets and copper/zinc gluconate tablets (both types of tablets contained magnesium stearate/stearic acid as a lubricant and microcrystalline cellulose as an emulsifier) were ground using an agate mortar and a pestle until a homogenous powder was formed; the powder was stored at 4 °C. For the determination of the total amount of the elements of interest (copper and zinc), the samples (0.2 g dry mass) were digested by microwave-assisted mineralization with a mixture of 5 mL of HNO_3_ and 3 mL of H_2_O_2_. The digest was diluted to a final volume of 10 mL with Milli-Q water. Further dilutions for ICP–MS analysis were prepared using 2 % nitric acid solution and 10 ng mL^−1^ of yttrium as an internal standard. The curves were linear in the investigated range of 5.0–120.0 μg L^−1^ with *r*^2^ above 0.999. Limits of detection (LOD) were calculated for standard deviations (SD) of 10 measurements for blank and were 0.5–1.4 μg L^−1^.

#### Soft extraction of metal species for SEC–ICP–MS analysis

Ground samples (0.05 g of dry powder) were sequentially extracted using an ultrasonic bath for 1 h with 2 mL of solvents in the following order: (1) 30 mM Tris–HCl (pH 7.4) to extract water-soluble element complexes and (2) 2 % SDS (sodium dodecyl sulfate) in water (pH 7.4) to extract hydrophobic proteins, which were suspected to reveal the ability to bind the investigated elements [26, 27]. The obtained solutions were centrifuged for 20 min at 10,000 rpm and 21 °C. The final supernatant was filtered with a 0.45-μm syringe filter (Sigma–Aldrich, Bellefonte, PA, USA); the first two drops were discarded and only the remaining part of the filtrate was injected onto the size exclusion column.

### In vitro simulation of gastrointestinal digestion for SEC–ICP–MS and μ-RPLC–ESI–MS/MS analyses

The in vitro digestion method was based on Luten et al.’s method [28] and modified for the Spirulina tablets studied. Thus, 2.5 mL of gastric juice (6 % w/v pepsin in 0.15 M NaCl, acidified with HCl to pH 1.8) was added to 0.07 g of the ground Spirulina tablets and to 0.05 g of the ground tablets containing zinc and copper gluconate and sonicated for 20 min in an ultrasonic bath. The mixtures were incubated in the thermostatic water bath for 3.5 h at 37 °C. After gastric digestion, the samples were divided into two groups. Samples from one group were centrifuged at 10,000 rpm for 20 min. Each supernatant henceforth referred to as “gastric extract” was filtered using 0.45-μm filters. Samples from the second group were subjected to intestinal digestion.

**Fig. 1:**
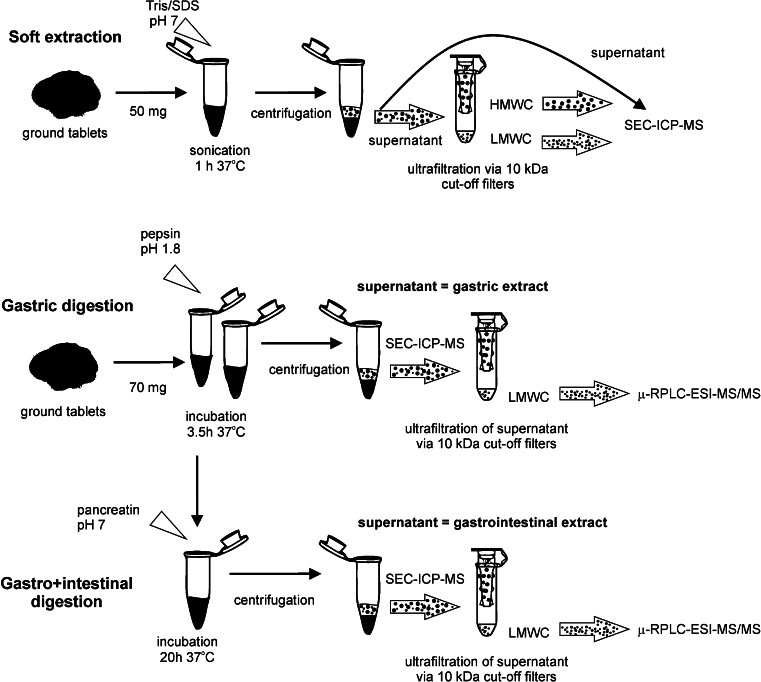
Analytical procedure for soft and enzymatic extraction of zinc and copper analysis

A 2.5 mL sample of intestinal juice (1.5 % w/v pancreatin in 0.15 M NaCl) was added to gastric digests and sonicated for 20 min in an ultrasonic bath. The mixtures were incubated in a thermostatic water bath for 20 h at 37 °C. After gastrointestinal digestion, the samples were centrifuged at 10,000 rpm for 30 min. Each supernatant obtained via centrifugation is henceforth referred to as “gastrointestinal extract”. Aliquots of the gastric and gastrointestinal extracts were filtered with a 0.45-μm syringe filter (Sigma–Aldrich, Bellefonte, PA, USA). Additionally, the samples were ultracentrifuged using 10-kDa cut-off filters to separate enzymatic proteins from small molecular weight compounds [29, 30]. The sample preparation procedure is summarized in Fig. 1. The efficiency of extraction enhanced by enzymatic digestion was estimated by establishing the amounts of elements in extracts relative to the total amount of elements obtained by mineralization (Table 1).

**Table 1:** Amounts of elements in the extracts of Spirulina tablets and Zn/Cu gluconate tablets

	Cu, μg g^−1^ (%)	Zn, μg g^−1^ (%)
Spirulina Pacifica		
30 mM Tris–HCl pH 7.4	2.9 ± 0.1 (20)	9.4 ± 0.4 (20)
2 % of SDS in water	3.5 ± 0.1 (24)	8.1 ± 0.3 (17)
Gastric digestion	8.2 ± 0.2 (56)	44.8 ± 1.1 (95)
Gastric and gastrointestinal digestion	13.2 ± 0.2 (90)	52.5 ± 1.7 (111)
Total amount of metal	14.5 ± 0.4	46.7 ± 0.7
Gluconate complexes		
Tris–HCl pH 7.4	1286 ± 35 (90)	13,263 ± 114 (55)
Gastric digestion	1376 ± 36 (96)	14,531 ± 134 (60)
Gastric and gastrointestinal digestion	1475 ± 45 (103)	15,148 ± 171 (62)
Total amount of metal	1579 ± 40	20343 ± 356

Results represent an average amount established for 3 samples; each measured 3 times; % of total amount of metal

It should be pointed out that two types of blank samples for gastric and gastrointestinal digestion procedures were investigated: (1) gastric and gastrointestinal juice for background subtractions during ESI–MS screening analysis, (2) gastric and gastrointestinal juice spiked before incubation with zinc and copper (10 μg mL^−1^) to verify the affinity of metal ions toward products of self-digestion.

## Results and discussion

### Total content of metals in Spirulina Pacifica and Zn/Cu gluconate tablets

The total amount of the elements studied in Spirulina tablets was established by means of ICP–MS as 14.5 μg g^−1^ (RSD 5.5 %) for copper and 46.7 μg g^−1^ (RSD 4.5 %) for zinc. It should be pointed out that besides zinc and copper, the contents of other microelements were also determined and the total amounts of magnesium (6702 μg g^−1^ (RSD 5.1 %)) and iron (2433 μg g^−1^ (RSD 6.5 %)) were estimated. The results are in agreement with the earlier reports by other authors [31]. The total amount of zinc and copper in copper/zinc gluconate tablets was also determined by the same method as 1579 μg g^−1^ (RSD 2.5 %) and 20,343 μg g^−1^ (RSD 3.5 %), respectively. The total amounts for zinc and copper obtained were in agreement with the specification provided by the producer of the supplements.

The contents of metals were also established for filtered supernatants obtained by extraction and enzymatic digestion of tablets containing cyanobacteria or gluconate complexes with copper and zinc. The extraction yields of metals obtained by different extraction methods and enzymatic digestion are presented in Table 1. Although copper gluconate and zinc gluconate are water soluble (100 g/L, pH 5.5–7.5), the recovery of copper was 90 %, whereas that of zinc was only 55 % with Tris buffer, even when a larger excess of solvent was used relative to the investigated powder. This effect can be explained by interaction of zinc complex with other components of the tablets (most probably hydrophobic stearic acid) leading to formation of insoluble zinc compounds. Enzymatic digestion improved the recovery of both metals in the case of gluconate tablets. Although the changes in zinc recovery are very low, they were established by ANOVA tests as statistically significant in comparison to those for Tris extraction (*p* = 0.00015 and *p* = 0.00005 for gastric and gastrointestinal digestion, respectively). Low improvement in zinc recovery using pepsin in acidic media excludes the formation of insoluble zinc hydroxides at pH greater than 7 during Tris extraction and supports the hypothesis about the presence of stable and insoluble zinc stearate in the investigated powder.

A different effect was observed for Spirulina tablets. Only 20 % of copper and zinc were water soluble. The presence of SDS did not improve the efficiency of extraction of either metal. Application of the gastric digestion protocol significantly improved the zinc recovery which reached 95 %. Such results indicate that the proteins are the main ligands binding zinc in Spirulina. In contrast to zinc, both steps of the digestion method were necessary to solubilize all miscellaneous species of copper. Hence, except for low molecular weight compounds or proteins, polysaccharides can be proposed as the ligands interacting with copper, because intestinal juice (rich in glycoside hydrolases) significantly improved the recovery of this metal in comparison to pepsin (*p* = 0.005).

### SEC–ICP–MS profiling of Tris and SDS extracts containing Cu/Zn compounds

**Fig. 2:**
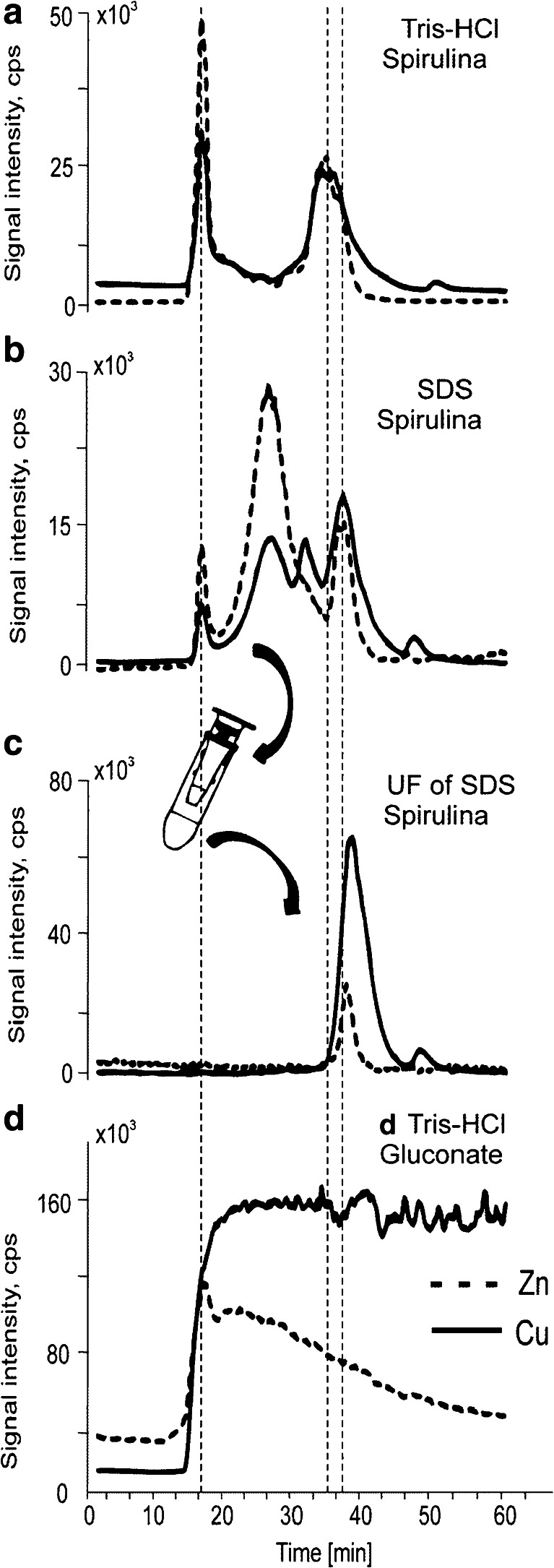
SEC–ICP–MS chromatograms obtained for supernatants of extracts of Spirulina tablets obtained with solutions of **a** 30 mM Tris–HCl (pH 7.4), **b** 2 % SDS in demineralized water, **c** ultrafiltrate of supernatant obtained with the same solution as in **b**, **d** 30 mM Tris–HCl (pH 7.4) extract of tablets containing Zn (*dotted lines*) and Cu (*solid lines*) gluconate

The Tris–HCl and SDS extracts of Spirulina were investigated by SEC–ICP–MS (Fig. 2a–c). For both investigated elements the SEC–ICP–MS chromatograms of Tris–HCl extracts showed two main peaks (Fig. 2a). The first peak at 15 min was obtained at the column’s exclusion limit corresponding to high molecular weight (HMW) compounds of at least 500 kDa. The second peak at 34 min corresponded to the fraction of copper and zinc complexes with molecular mass 17 ± 10 kDa. For copper one small additional peak at 50 min was observed, corresponding probably to low molecular weight complexes. The chromatograms obtained for SDS extracts were much more complex. Additional new peaks (especially for copper) corresponding to compounds with molecular mass between 25 and 60 kDa were obtained. For zinc only a single but wide peak was obtained at 26 min (25 kDa).

The peak obtained at 15 min in the void volume of the column corresponding to complexes with molecular mass greater than 500 kDa was the most intense for Tris extracts. As high molecular weight proteins were more expected to occur in SDS extracts, the nature of the corresponding compound was clarified. The extracts were ultrafiltrated using 10-kDa-cut-off filters and both obtained fractions: proteins and ultrafiltrates were analysed again by SEC–ICP–MS. The chromatograms obtained for the fractions containing adducts of Cu and Zn with proteins larger than 10 kDa consisted of peaks eluting in the range of 15–30 min, including the peak at 15 min (not shown). The chromatograms for ultrafiltrates consisted of one main peak at 39 min obtained for both Tris and SDS extracts (Fig. 2c). The presence of hydrophilic complexes larger than 500 kDa can be explained by the ability of metal ions to form agglomerates with both proteins and low molecular weight bioligands, which has already been reported for metal complexes with glutathione [20]. In particular, the addition of SDS to extractant solution did not improve the recovery of the metals, but was mainly responsible for changes in the chromatographic profile (decreased peak at 15 min and new peaks at 26 and 32 min (Fig. 2b), probably related to denaturation of proteins). By comparison of the total peak areas obtained for ultrafiltrates and the samples before ultrafiltration, the relative amount of low molecular weight complexes containing copper was established as 49 % and 21 % in Tris and SDS fraction, respectively. As far as zinc complexes are concerned, low molecular weight compounds comprise only 27 % and 8 % of Tris and SDS fraction, respectively. Such a difference in recovery can be explained by the lower stability of zinc complexes in comparison to that of copper ones.

The epoxy bridges present in the polydextran stationary phase can interact with metal ions leading to partial decomposition of complexes or metal exchange. This process can influence metal recovery from the stationary phase leading to longer retention of compounds or loss of signal in the ICP–MS detection. Such behaviour was observed for the tablets containing zinc and copper gluconate dissolved in Tris–HCl buffer. The chromatograms showed small peaks at times related to void volume of the column followed by a high level of baseline (Fig. 2d), even when the concentration of zinc and copper was two orders higher than in the extracts of Spirulina. Moreover, no additional chromatographic peak was observed even when the analysis was prolonged until 90 min. Additionally, metal recovery from the SEC column was established by ICP–MS (as the ratio of metal injected onto the column to that in the collected eluate) as 42 % for copper and 31 % for zinc. Both recoveries increased up to 59 % and 43 % when 10 mM of NaCl was present in the solution of the mobile phase and the time needed to obtain the level of baseline before injection was shortened significantly. On the other hand, the recoveries of Zn and Cu from the SEC column obtained for Tris extracts of Spirulina were 99 % and 105 %, respectively, leading to the conclusion that the stability of the gluconate complexes with the investigated metals is significantly lower than that of the metal adducts with bioligands present in the cyanobacteria.

Stability of high molecular weight complexes formed by proteins and peptides may differ significantly from that of low molecular ones, which is why mixtures of histidine (low molecular weight compound known to bind zinc and copper) with the investigated metal ions were analysed by SEC–ICP–MS. The chromatograms showed one main peak corresponding to copper and zinc complexes (see Electronic Supplementary Material Fig. S1). It should be pointed out that a significant molar excess of histidine to zinc (10:1) was necessary to observe peaks in the chromatogram of zinc.

### SEC–ICP–MS characteristics of gastric and gastrointestinal digests of cyanobacteria and gluconate complexes with zinc and copper

**Fig. 3:**
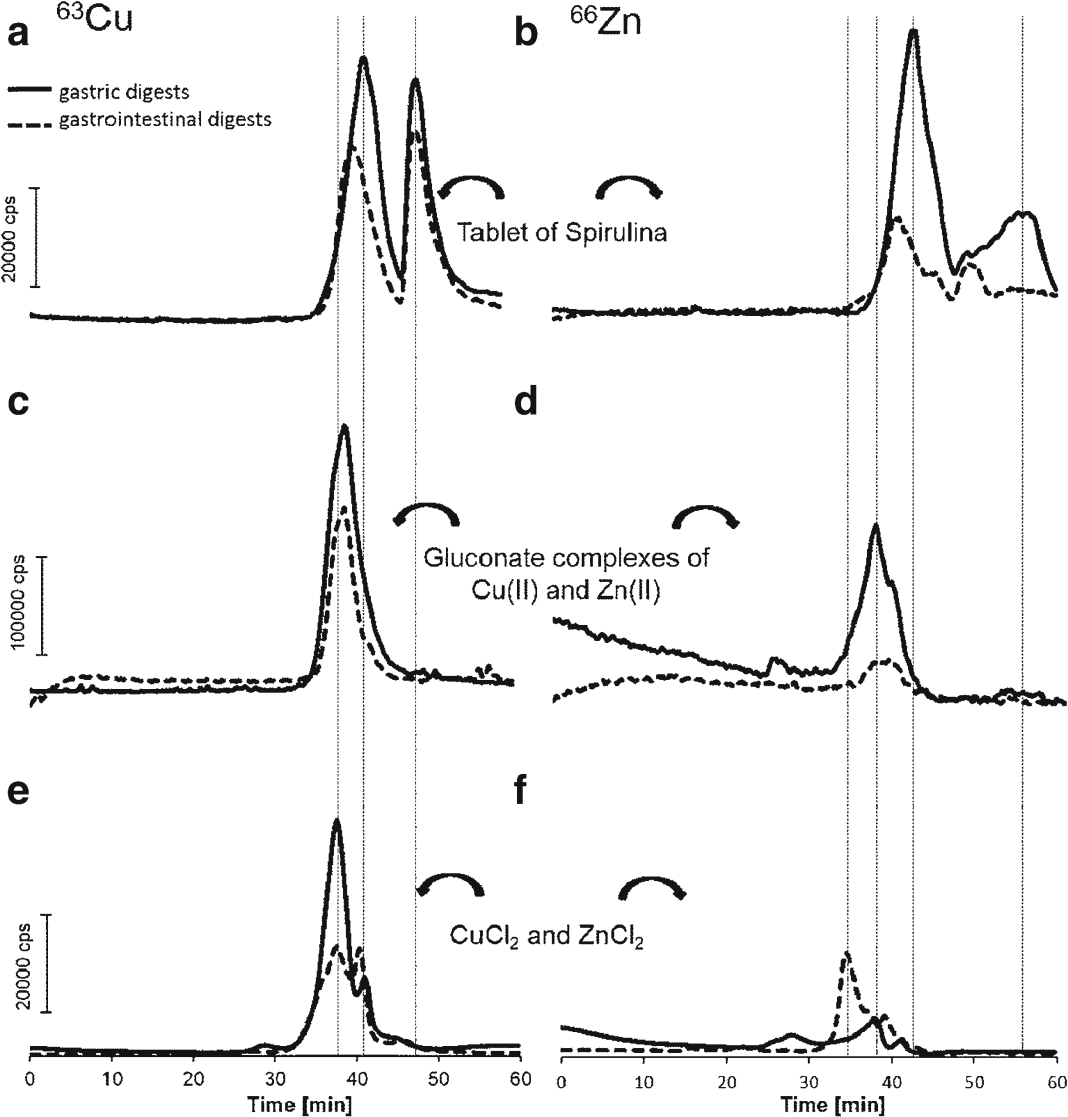
SEC–ICP–MS chromatograms of gastric (*solid line*) and gastrointestinal digests (*dotted line*) of **a**, **b** Spirulina; **c**, **d** gluconate complexes with Zn and Cu and **e**, **f** inorganic form of copper and zinc (10 and 40 μg/g, respectively). *Left column* **a**, **c**, **e** chromatograms are presented for copper; *right column* **b**, **d**, **f** chromatograms are presented for zinc

Enzymatic extracts of powdered tablets were examined by SEC–ICP–MS using the same elution conditions as for Tris and SDS extracts. The chromatograms of Spirulina extracts obtained for ^63^Cu after digestion with both pepsin and pancreatin were the same and showed two similar peaks (*t*_R_ = 41_Cu_/42_Zn_ and 47_Cu_/57_Zn_ min) (Fig. 3a,b). However, in the chromatograms, the second chromatographic peak of zinc was significantly lower than the first. Similar chromatograms were obtained for proteins extracted from cyanobacteria and subjected to gastric digestion (see Electronic Supplementary Material Fig. S2).

The chromatograms obtained for copper and zinc gluconate revealed only one peak with a maximum at about 37 min (Fig. 3c,d). This was observed due to metal binding by enzymatic proteins (pepsin, ~35 kDa expected at 29 min and trypsin, ~23 kDa expected at 35 min) or their self-digestion products; however, it was impossible to distinguish between these because similar retention times were obtained for inorganic forms of copper and zinc subjected to gastric and gastrointestinal digestion (Fig. 3e,f) or a complex of a metal ion with a single amino acid (see Electronic Supplementary Material Fig. S2). On the other hand the ability of enzymatic proteins to interact with metal species and improve their bioaccessibility has already been reported for cobalamins [25].

As the stability of Cu/Zn gluconate complexes is rather low, uncontrolled competition between metal ions or ligands present in tablets (stearic acid or cellulose) can influence their bioaccessibility. A 0.5 mL solution containing zinc (100 μg mL^−1^) and molar excess of histidine (1:10) was subjected to gastric and gastrointestinal digestion and the yields of the extractions were established as 79 % and 88 %, which is almost 30 % higher than those achieved for gluconate tablets (Table 1). In the second step, the same solution, but with 1 mg stearic acid, was subjected to the same digestion protocol, i.e. to gastric and gastrointestinal digestion, and yields for zinc were 58 % and 62 %, respectively. It can be concluded that zinc ions show high affinity toward stearic acid, a highly hydrophobic compound, which can deteriorate metal bioaccessibility. In the case of Spirulina tablets, an excess of peptides and proteins ensures that metal ions are still bound to bioligands, thereby improving the effectiveness of metal accumulation even in the presence of stearic acid, which is also used the formulation of tablets from Spirulina powder. Therefore, the kind of peptides/amino acids involved in copper and zinc binding is of particular interest.

### Identification of copper/zinc species by μ-HPLC–ESI–MS/MS: gastric peptide map

The gastric and gastrointestinal mixtures after ultrafiltration were subjected to μ–HPLC–ESI–MS/MS. The analysis was carried out by recording the ions in the *m/z* range from 50 to 600, 580 to 1000, and 980 to 1500 in both negative and positive ion modes. The mass spectra recorded for each chromatographic peak were carefully searched for signals with isotopic patterns corresponding to mono and doubly charged Cu_*n*_, Zn_*n*_ and Cu_*n*_/Zn_*n*_ complexes with agreement higher than 80 %, where *n* = 1–3. In the next step, two kinds of blank samples were analysed: (1) enzymatic mixtures used for both steps of the digestion protocol to check for desorption of metal ions from the stationary phase of the chromatographic column and (2) the same mixtures but with metal ions added before digestion to indicate metal complexes formed by enzymatic proteins and their digestion products.

**Fig. 4:**
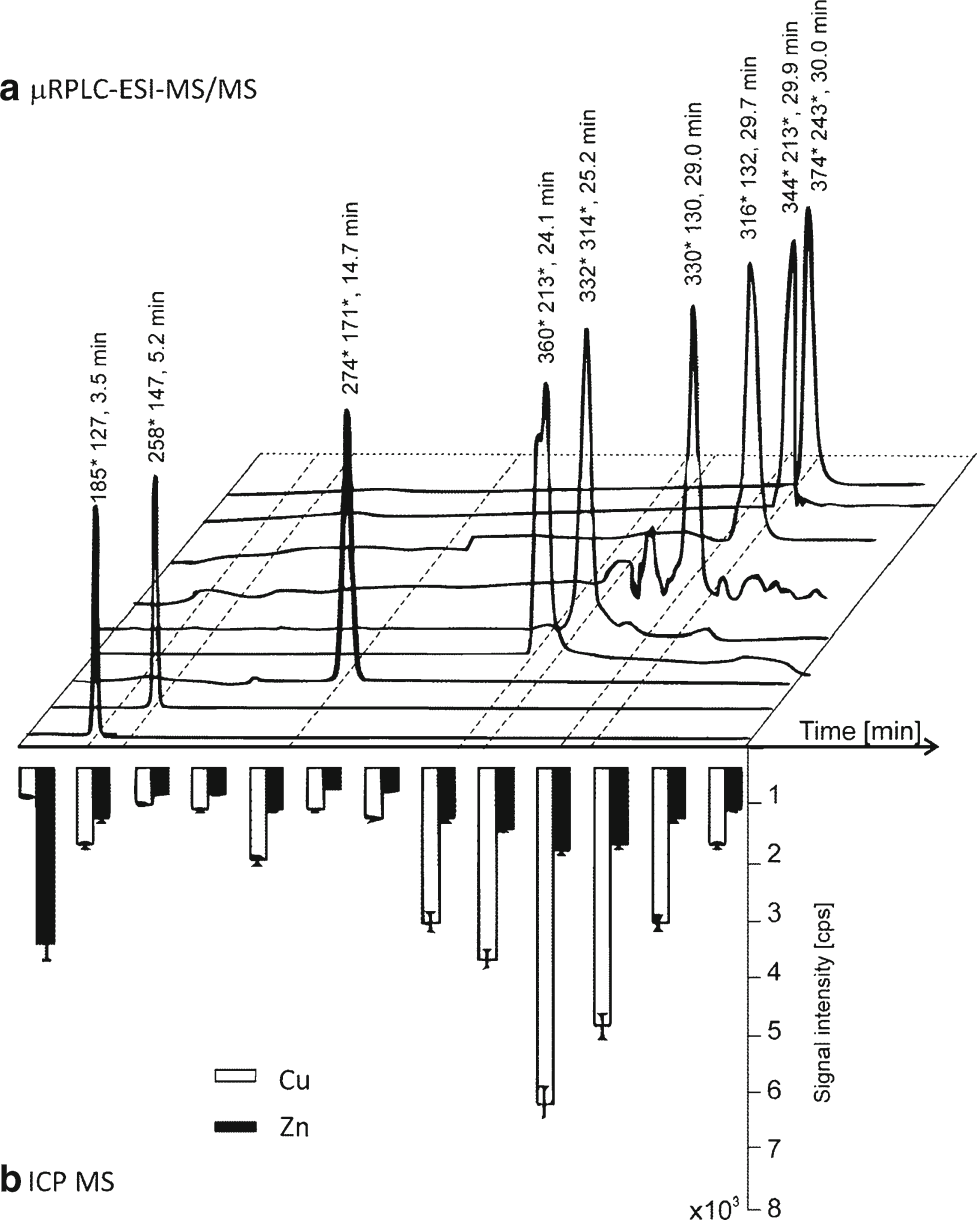
μ-HPLC–ESI–MS/MS chromatograms of Spirulina tablets after gastrointestinal digestion registered in positive ion SCAN mode (**a**) and the signal intensities of zinc and copper established by ICP–MS in fractions collected every 3 min (**b**). Chromatograms (**a**) were extracted for selected product ions, see Table 2 for details

After examination of the mass spectra, the signals with specific isotopic profiles were found only in the Spirulina digests. These were only ions with single charge corresponding to complexes with one copper atom. The chromatograms obtained for endogenic copper complexes (nine different signals, which were not observed for any of the blank samples) are presented in Fig. 4. The chromatogram can be divided into three parts with elution of (1) polar compounds at 3.5 and 5.2 min, (2) one less polar compound at 14.3 and (3) hydrophobic compounds with retention in the range 24–30 min (Fig. 4). It is possible that the complexes formed with zinc during the digestion process are not stable enough to survive the reversed-phase chromatographic process in acidic conditions. On the other hand, when ammonium formate was used (pH 4.5) the signals with copper and zinc isotopic profiles were not detected. It means that lower pH is required to obtain detectable signals by ESI–MS. It is possible that acidic media prevent decarboxylation combined with metal release from the complex in the ES ionisation chamber, which has already been reported by other authors [18].

With the aim of verifying the presence of zinc in the eluate, fractions (15 μL during 3 min each) from the capillary column were collected five times and were diluted by addition of 250 μL of formic acid solution for ICP–MS analysis. It was found that copper was present in most of the fractions and corresponded to chromatographic peaks selected by ESI–MS. The same was observed for zinc but intensities were lower and the highest signal was observed in the void volume of the column (Fig. 4a). Lower signal intensities were also observed for a histidine complex with zinc in comparison to copper (not shown). It can be concluded that zinc complexes are present in enzymatic digests of Spirulina but their low stability and large number of stable isotopes (64–49 %, 66–28 %, 67–4 %, 68–19 %, 70–1 %) preclude their identification by molecule-specific MS.

In order to identify the ligands that can bind copper ions, the fragmentation was carried out for pairs of quasi-molecular ions following the isotopic profile of copper (63–69 % and 65–31 %). The first mass spectra for product ions obtained at 3.5 min for both parent positive ions at *m/z* 185 and 187 showed one specific signal at *m/z* 69 (without metal). The odd *m/z* value and positive charge indicate the presence of an even number of nitrogen atoms, which is consistent with the presence of an imidazole ring typical of histidine and histamine. The *m/z* value of the quasi-molecular ion does not correspond to any of these molecules and the number of signals is too limited to establish the structure. The most intense signal observed in the mass spectra was at *m/z* 127 assigned to the loss of 58 and 60 units from the parent ion, which is not equivalent to isotopes of copper or zinc. However, if the histidine derivative obtained by the loss of an oxygen molecule at 155 − 32 = 123 amu and copper are considered (123 + 63 − 2 = 184 amu, [M + H]^+^ at *m/z* 185), the histidine residue bound to copper can be proposed as the most probable digestion product of the Cu–protein adducts. The agreement between the theoretical and experimental isotopic patterns obtained in MS mode was 97 %. The second fragmentation mass spectrum was obtained for *m/z* 258 at 5.2 min. It consists of many signals obtained after the loss of copper. The differences between signals, such as 17 and 46 parallel to 18 and 45, indicate the presence of amine and hydroxyl groups bound to an ethyl moiety (Table 2). The difference of 93 and 95 between signals at *m/z* 240 and 147 can correspond to the loss of copper and formaldehyde, which indicates involvement of a carboxylic group in metal binding. Analysis of all the signals (Table 2) indicated the complex of copper with peptide Asp-Gly as the most probable candidate, especially as the proposed peptide is polar owing to the presence of at least two carboxylic groups.

**Table 2:** Copper species observed in μ-HPLC–ESI–MS/MS spectra of Spirulina tablets after simulation of gastric and gastrointestinal digestion

No.	*t*_R,_min	[M + H]^+^/[M − H]^−^, M Proposed structure	Pepsin/pancreatin	Product ions of [M + H]^+^ or [M − H]^−^
1	3.5	185^*^/ND, 184 derHis=Cu	+/−	(ESI+) 69 (Imd), 127 (Imd + C_3_H_7_OH)
2	5.2	258^*^/ND, 257. 1 Asp-Gly=Cu + 3H_2_ − 2H_2_O	+/−	(ESI+) 70 (C_4_H_7_N), 84 (C_4_H_5_NO), 129 (84 + CH_3_CH_2_NH_2_), 130 (84 + CH_3_CH_2_OH), 147 (129 + H_2_O or 130 + NH_3_), 240* (147 + Cu + HCOH), 258* (240* + H_2_O)
3	14.7	274*/ND, 273.1 Pro-Pro=Cu − 2H_2_O	+/+	(ESI+) 72 (Prl), 173* (Pro=Cu − 3H_2_), 199* (Pro-Cu-CO)
4	24.1	360*/358*, 359.1 Phe-Cu-Asp – H_2_O	+/−	(ESI+) 86 (C_4_H_7_NO), 213* (Phe-Cu − H_2_O + H_2_), 247* (Phe-Cu + H_2_O)
5	25.2	332*/330*, 331.1 His-Asp=Cu − 2H_2_O	+/−	(ESI+) 86 (C_4_H_7_NO), 134* (C_3_H_6_N_2_=Cu), 201* (His=Cu − H_2_O + CH_4_), 314* (His-Asp=Cu − H_2_O)
6	29.0	330*/328*, 329.1 Ser-Tyr=Cu − 2H_2_O	+/−	(ESI -) 130 (C_6_H_9_NO_3_), 284* (Ser-Tyr=Cu − CO_2_)
7	29.7	316*/ND, 315.1 Val-His=Cu − 2H_2_O	+/+	(ESI+) 86 (C_4_H_7_NO), 132 (Val + H_2_O − 2H_2_), 157* (C_2_H_3_NO=Cu + 2H_2_O), 185* (His=Cu − 2H_2_O + 2H_2_), 203* (His=Cu − H_2_O + 2H_2_)
8	29.9	344*/342*, 343.2 Thr-Tyr=Cu − 2H_2_O	+/−	(ESI−) 130 (C_6_H_9_NO_3_), 185 (300* − H_4_CO_3_Cu), 211* (300* − C_3_H_8_NO_2_), 300* (344* − CO_2_)
9	30.0	374*/372*, 373.1 Met-Tyr=Cu − 2H_2_O	+/−	(ESI+) 243* (Tyr=Cu − H_2_O)

*Signals corresponding to the second isotope of copper/zinc (2 units higher)

*ND* not detected, *M* experimental monoisotopic mass established with 50–300 ppm accuracy, *derHis* histidine derivative obtained by loss of oxygen, *Imd* imidazole, *Prl* pyrrolidine

The next fragmentation was carried out for the parent ions at *m/z* 274 and 276 at 14.7 min. The most important signal at *m/z* 72 corresponds to a pyrrolidine ring typical of a proline amino acid responsible for the longer retention time and the signal at *m/z* 173 was assigned to the loss of proline from the complex of Pro-Pro peptide with copper.

More hydrophobic compounds were found to be more stable and for some of them the number of fragmentation ions with copper increased. The first mass spectrum was obtained at 24.1 min for parent ions at *m/z* 360 and 362 and the most characteristic value *m/z* 213 was concluded to correspond to the residue of phenylalanine (−H_2_O) bound to copper. Moreover, a phenyl group can be additionally responsible for the longer retention time. For the parent ions of *m/z* 332 and 334 which fragmented at 25.2 min, the most specific signals were obtained at *m/z*132 and 134, which correspond to copper bound to an imidazole ring indicating the presence of a His residue. The lowest signal at *m/z* 86 was assigned to the loss of copper and imidazole group. Another residue can be aspartic acid, formed as a result of the loss of three water molecules upon fragmentation (see Table 2). The signal at *m/z* 86 was also obtained for the parent ions of *m/z* 316 and 318 at 29.7 min for peptide Val-His bound to copper.

**Fig. 5:**
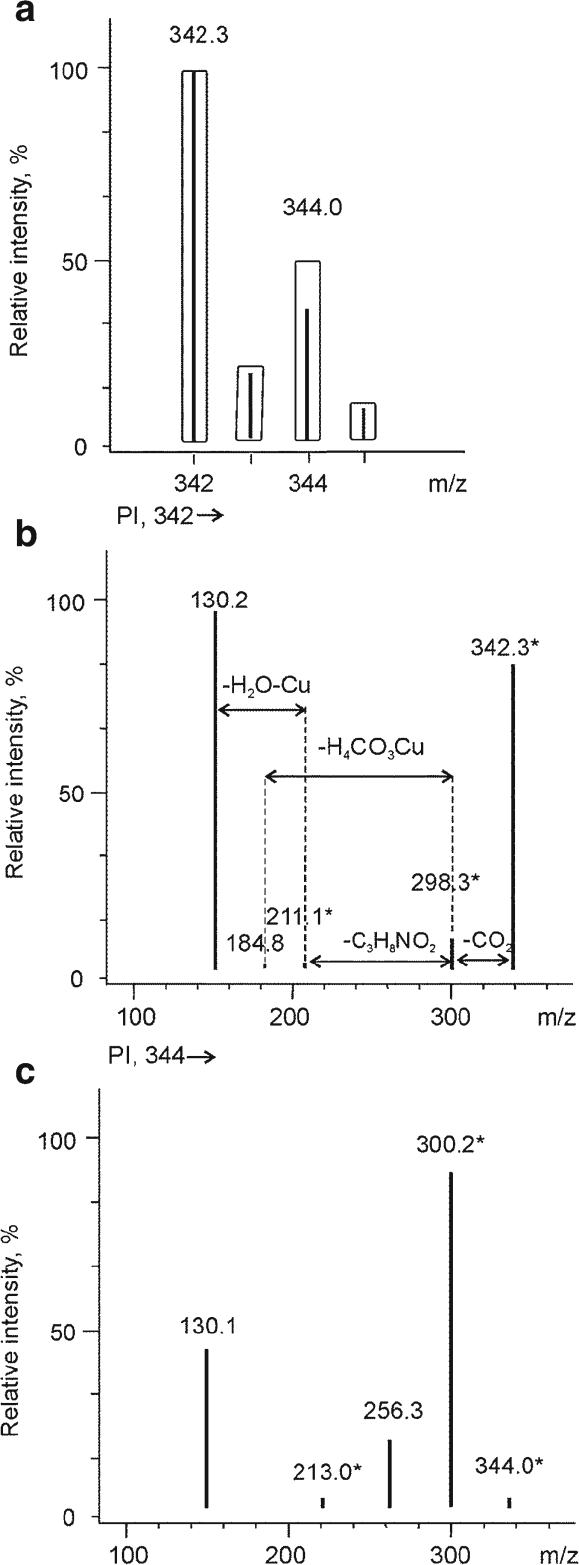
Mass spectra obtained by means of μ-HPLC–ESI–MS/MS in negative ion mode and extracted at 29.9 min in the SCAN mode (**a**) for comparison of theoretical (*bars*) and experimental isotopic profile (*lines*). The product ion (PI) mass spectra were obtained for the two most intense signals at **b** *m/z* 342 and **c** *m/z* 344 following isotopic profile of copper. Signals with an *asterisk* correspond to molecules containing metal (see also Table 2)

Another interesting group of signals appeared at 29.0, 29.9 and 30.0 min at *m/z* 330, 344 and 374, respectively. It should be stressed that the most intense signals were observed in the negative ion mode at *m/z* 328, 342 and 372 (see Fig. 5 for the interpretation of mass spectra obtained for the signal at *m/z* 342). The molecular mass and fragmentation ions indicated the presence of a tyrosine residue containing a phenyl group, which can be easily deprotonated. The signals were identified as corresponding to copper complexes with the peptides Ser-Tyr, Thr-Tyr and Met-Tyr, respectively. All the fragmentation signals are presented in Table 2.

It should be pointed out that longer retention times are strictly related to the presence of aromatic or heterocyclic rings in the structure of amino acids which are responsible for hydrophobic interactions with the ODS stationary phase. Another reason for such hydrophobic character of the complexes can be strong affinity of copper toward amine groups, thereby preventing protonation of amine groups in acidic media. However, the solubility of these complexes is much higher than that of the complexes formed with polyphenols that are responsible for the lower bioavailability of iron in plants [32]. It should be mentioned that almost all signals were obtained as a result of simulation of gastric digestion with pepsin. Copper ions as well as iron and nickel have already been reported to be bound by pepsin even in the acidic media [33] leading to partial deactivation of pepsin. It should be stressed that low signals at *m/z* 342 and 340 (S/N about 10 and 3 in positive ion mode, respectively) were also observed at 29.9 min after pepsin self-digestion in the presence of copper ions. Moreover, another pair of signals was also observed in the spectrum of the blank sample subjected to pepsin digestion protocol and spiked with copper: *m/z* 377 and 326 at 4.1 and 27.8 min in positive ion mode, respectively. This confirms the ability of pepsin self-digestion products to bind metal ions. Unfortunately, the intensity of these signals was too low to obtain fragmentation ions necessary for identification of the compounds.

The short peptides proposed with well-defined affinity towards copper ions contain residues of amino acids such as cysteine, histidine and methionine. These amino acids have already been reported by other authors to bind copper ions [34]. However, for pepsin digests the involvement of the other amino acids such as aspartic acid, phenylalanine and tyrosine was found to be dominant.

Although zinc was found by SEC–ICP–MS to form complexes with proteins or digestion products of pepsin and pancreatic enzymes, only one signal was found in μ-RPLC–ESI–MS/MS (histidine complex, *m/z* 186), probably as a result of low stability of the complexes. However, the involvement of proteins and their components in enhancement of zinc bioaccessibility cannot be excluded.

## Conclusions

In this study, the bioaccessibilities of copper and zinc from the cyanobacteria extracts and from zinc and copper gluconate were compared for the first time by ICP–MS and SEC–ICP–MS methods. A new μ-RPLC–ESI–MS/MS method was developed to obtain specific gastric peptide maps for identification of copper–amino acids complexes in the extracts of Spirulina tablets after in vitro simulation of gastric and gastrointestinal digestion.

It was found that blank samples with added metal ions are required to indicate metal complexes formed with self-digestion products of enzymatic proteins.

It was demonstrated by SEC–ICP–MS that zinc and copper complexes can be formed by cyanobacteria-originating proteins and peptides. It was proved that the presence of stearic acid deteriorates the bioaccessibility of zinc. It is possible that proteins prevent the formation of insoluble zinc stearate during formation of tablets from cyanobacteria; however, this aspect requires additional investigation.

The μ-HPLC–ESI–MS/MS results obtained show that the most stable complexes are those between copper and amino acids and peptides obtained via gastric digestion (with pepsin in acidic media) of copper-proteins from cyanobacteria.

Apart from histidine which has already been reported to bind copper ions, other interesting amino acids such as aspartic acid, phenylalanine, proline and tyrosine were shown to be able to form complexes of different hydrophobicity, solubility and bioavailability.

## Electronic supplementary material

ESM 1 (PDF 316 kb)
