# Supplementary material for: Comparison of copper and zinc in vitro bioaccessibility from cyanobacteria rich in proteins and a synthetic supplement containing gluconate complexes: LC–MS mapping of bioaccessible copper complexes
Source: Anal Bioanal Chem. 2015 Nov 23;408:785–95. doi: 10.1007/s00216-015-9162-8 (PMC4709381; doi:10.1007/s00216-015-9162-8)
Supplement: Supplementary file 1 — (PDF 316 kb) [file 216_2015_9162_MOESM1_ESM.pdf]

Analytical and Bioanalytical Chemistry

Electronic Supplementary Material

Comparison of copper and zinc in vitro bioaccessibility from cyanobacteria rich in proteins and a synthetic supplement containing gluconate complexes: LC-MS mapping of bioaccessible copper complexes

Justyna Wojcieszek, Katarzyna Witkoś, Lena Ruzik, Katarzyna Pawlak

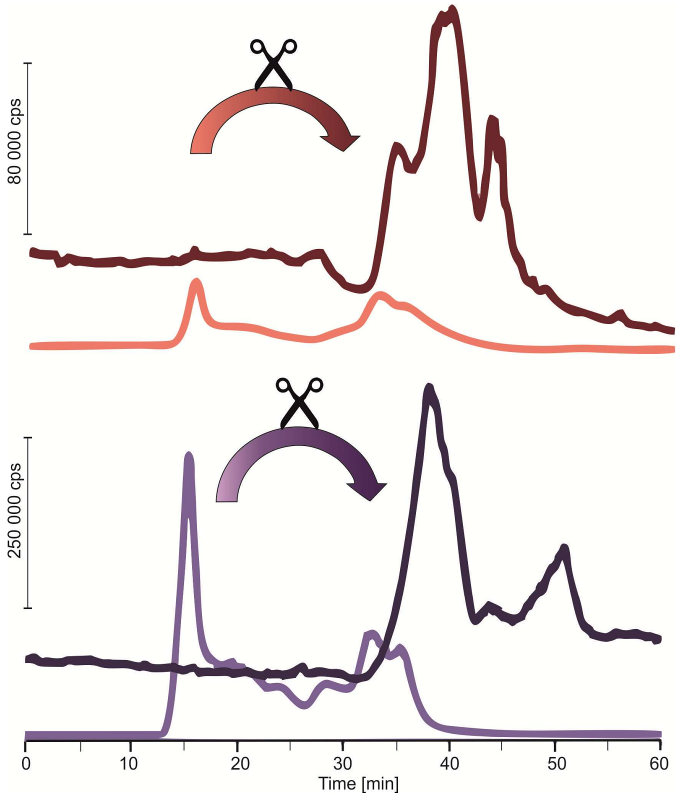

Fig. S1 SEC-ICP-MS chromatograms of protein fraction of Spirulina obtained by ultrafiltration via 10 kDa cut-off filters (light red for Cu-protein and light violet for Zn-protein fraction) subjected to pepsin digestion (dark red for Cu-compounds and dark violet for Zn-compounds).

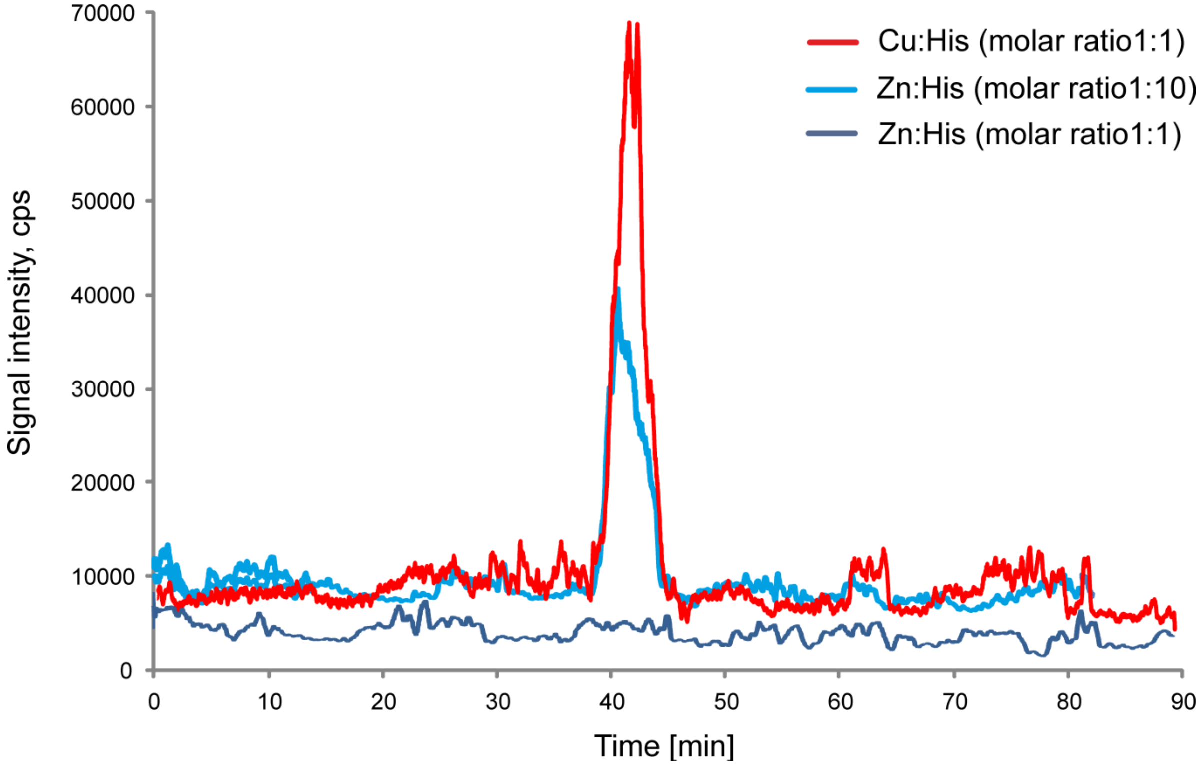

Fig. S2 SEC-ICP-MS chromatograms obtained for mixtures of histidine with zinc at molar ratio 1:1 and 10:1 and copper in molar ratio 1:1. The concentration of metal was 1 µg g^{-1}

Table S1 Operational parameters for HPLC, ICP-MS and ESI-MS

Settings	
**ICP-MS**	**Agilent 7500a**
RF Power	1350 W
Plasma, auxiliary and nebulizer gas flow	15.0, 1.0 and 1.05 L min^{-1}
Cones	Sampler – Ni, Skimmer – Ni
Monitored isotopes	^{59}Co, ^{63}Cu, ^{65}Cu, ^{66}Zn, ^{67}Zn, ^{68}Zn
Dwell time	0.1 ms
**HPLC separation**	
pump	Agilent 1100
Column	Superdex 200 (10 x 300 mm x 10 µm) – GE Healthcare Life Sciences
Mobile phase	30 mM Tris-HCl buffer (pH 7.4)
Elution program	isocratic
Flow	0.5 mL min^{-1}
Injection volume	100 µL
Column temperature	24˚C
Pump	Agilent 1200 Series
Column	Zorbax SB C18 (3.5µm 0.3x150mm)
Injection volume [µL]	0.1
Flow rate[µL min $^{-1}$]	5
Eluents	A: mixture water – methanol (95:5 v/v) with 0,1% formic acid
	B: mixture methanol – water (95:5 v/v)
Gradient program	Time, min%B
	0 0
	8 0
	23 90
	35 90
**ESI-MS/MS detection**	
Detector	Agilent 6460 Triple Quad LC/MS with JetStream technology
Polarity	positive, negative
Mode	SCAN, PI
Ionization voltage [V]	2500(PI), 1500(NI)
Nebulizer pressure [psi]	55
Gas temperature [°C]	300
Gas flow [L min^{-1}]	8
Sheath gas flow[L min^{-1}]	6
Sheath gas temperature [°C]	300
Mass range, m/z	50-600, 580-1000, 980-1500
Collision energy, eV	5-30
